# Different levels of humoral immunoreactivity to different wheat cultivars gliadin are present in patients with celiac disease and in patients with multiple myeloma

**DOI:** 10.1186/1471-2172-10-32

**Published:** 2009-05-31

**Authors:** Aleksandra Konic-Ristic, Dejan Dodig, Radmilo Krstic, Svetislav Jelic, Ivan Stankovic, Aleksandra Ninkovic, Jelena Radic, Irina Besu, Branka Bonaci-Nikolic, Njegica Jojic, Milica Djordjevic, Dragan Popovic, Zorica Juranic

**Affiliations:** 1 Institute of Bromatology, Faculty of Pharmacy, Belgrade, Serbia; 2 Maize Research Institute, Zemun, Serbia; 3 Institute of Digestive Diseases, Clinical Center of Serbia, Belgrade, Serbia; 4 Institute for Oncology and Radiology of Serbia, Belgrade, Serbia; 5 Institute of Allergology and Immunology, Clinical Center of Serbia, Belgrade, Serbia; 6 Metabolic Department, Center for Gastroenterology and Hepatology, Zvezdara University Clinical Center, Belgrade, Serbia

## Abstract

**Background:**

Immunity to food antigens (gliadin, cow's milk proteins) is in the centre of the attention of modern medicine focused on the prevention of diseases, prevention which is based on the use of appropriate restriction diet. Detection of the enhanced levels of the immune reactions to antigen(s) present in food is from this point of view of great importance because there are reports that some of health disturbances, like celiac disease (CD) and some premalignant conditions, like monoclonal gammopathy of undetermined significance (MGUS), were vanished after the appropriate restriction diets.

It is well known that gliadin is toxic to small bowel mucosa of relatively small population of genetically predisposed individuals, who under this toxic action develop celiac disease (CD). As the quantity of immunogenic gliadin could vary between different wheat species, the first aim of this work was to determine the percentage of immunogenic gliadin in ten bread wheat cultivars and in three commercially grown durum wheat cultivars. The second part of the study was initiated by results of previous publication, reporting that sera of some of multiple myeloma (MM) patients showed the presence of elevated levels of anti-gliadin IgA, without the enhanced levels of anti-gliadin IgG antibodies, determined with commercial ELISA test. It was designed to assess is it possible to reveal is there any hidden, especially anti-gliadin IgG immunoreactivity, in serum of mentioned group of patients. For this purpose we tested MM patients sera, as well as celiac disease (CD) patients sera for the immunoreaction with the native gliadin isolated from wheat species used for bread and pasta making in corresponding geographic region.

**Results:**

Gliadin was isolated from wheat flour by two step 60% ehanolic extraction. Its content was determined by commercial R5 Mendez Elisa using PWG gliadin as the standard. Results obtained showed that immunogenic gliadin content varies between 50.4 and 65.4 mg/g in bread wheat cultivars and between 20 and 25.6 mg/g in durum wheat cultivars.

Anti-gliadin IgA and IgG immunoreactivity of patients' sera in (IU/ml) was firstly determined by commercial diagnostic Binding Site ELISA test, and then additionally by non-commercial ELISA tests, using standardized ethanol wheat extracts -gliadin as the antigen.

In both patients groups IgA immunoreactivity to gliadin from different cultivars was almost homogenous and in correlation with results from commercial test (except for one patient with IgA(λ) myeloma, they were more then five times higher). But, results for IgG immunoreactivity were more frequently inhomogeneous, and especially for few MM patients, they were more then five times higher and did not correlate with results obtained using Binding Site test.

**Conclusion:**

Results obtained showed different content of immunogenic gliadin epitopes in various species of wheat.

They also point for new effort to elucidate is there a need to develop new standard antigen, the representative mixture of gliadin isolated from local wheat species used for bread production in corresponding geographic region for ELISA diagnostic tests.

## Background

It is well known that gliadin is directly or indirectly through immune mediated reactions, toxic to small bowel mucosa of relatively small population of genetically predisposed individuals who under this toxic action develop celiac disease (CD). These patients need to eat food without gluten, i.e., they need to be on gluten free diet (GFD). Therefore very reliable tests are needed to determine is the content of gliadin really below the accepted value (20 mg/kg). As gliadin isolated from various species used as the antigen may have different immunogenicity [1] that fact could be a problem in the immunological tests used for determination of gliadin content in food; *i.e*., results may greatly depend on the origin and type of gliadin that was used for calibration. In the aim to overcome this analytical problem, "prolamin working group" developed a PWG gliadin which represents protein fraction soluble in 60% ethanol from the mixture of twenty-eight wheat cultivars grown in Great Britain, France and Germany [1-6]. This gliadin is recommended for use as the standard antigen in immunological techniques for determination of gluten content in food.

At the same time, it was very important to standardize anti-gliadin antibodies that should be used in immunological tests for determination of gliadin content. In the recent time few monoclonal or polyclonal anti-gliadin antibodies were developed. Commercial kits often used polyclonal antibodies developed against wheat gliadin, or a monoclonal antibody made against ω-gliadins from the Australian wheat cultivar 'Timgalen'. There were also other antibodies developed in different laboratories such as monoclonal antibody PN3, raised against a synthetic peptide equivalent to the amino acids 31–49 of α-gliadin, *i.e*. the sequence of toxic peptide of α-gliadin, which has been shown to cause mucosal damage to the small bowel of celiac patients [7,8]. The other polyclonal rabbit anti-gliadin antibody made for the same proposes was prepared against α-gliadin, isolated from the 'Kanzler' and other German wheat cultivars. At the present time the greatest attention has monoclonal antibody R5, developed against a secalin extract, which recognizes the potential celiac-toxic epitope QQPFP, which occurs repeatedly in α-, γ- and ω-gliadins, hordeins and secalins, of wheat, barley and rye to a similar degree. Applied in a sandwich ELISA, this antibody showed identical calibration curves for PWG- and Sigma-gliadin [6]. This antibody is now recommended by Codex Alimentarius [9,10] as an antibody which has to be used in sandwich ELISA method for determination of gluten content in gluten free food. Today there are commercial kits available for determination of gluten content, that include R5 antibody as anti-gliadin antibody and PWG gliadin for calibration (Ridascreen, R-Biopharm, Germany).

The most toxic α-gliadin, mainly from the 6D genome of wheat, is the major contributor in both activating the adaptive immune response and the innate immune response. Tetraploid wheat (*T. durum*) do not contain the D genome. This wheat is hence already devoid of the known immunogenic sequences of α-gliadins [11]. Molberg *et al*. [12] reported that several wheat lacking the D genome also lack the immunodominant 33-mer, but, they did not extend their conclusion to all durum wheat.

Therefore the first aim of this work was to determine the percentage of immunogenic gliadin isolated from ten bread wheat cultivars and three durum wheat cultivars grown commercially in Serbia and neighbouring countries.

Testing sera for IgA or IgG immunoreactivity to gliadin is usually one of the first steps in the complex process of diagnosing gluten intolerance, because it is well known that antibodies to native gliadin sequences are present in patients with celiac disease. This, second part of the study, was based on results of previous publication reporting that sera of some of multiple myeloma (MM) patients showed the presence of elevated levels of anti-gliadin IgA- without the enhanced levels of anti-gliadin IgG immunoglobulins, determined on commercial ELISA test. It was designed to assess is it possible to reveal is there any hidden, especially anti-gliadin IgG immunoreactivity, in serum of mentioned group of patients. For this purpose we tested MM patients' sera as well as celiac disease (CD) patients' sera for the antigliadin immunoreactivity using commercial ELISA test (Binding Site, Birmingham U.K), as well as home made ELISA tests using commercially available gliadin (alcohol soluble protein fraction from crude gliadin products of Sigma, and the native gliadin isolated from wheat species used from bread making in corresponding geographic region as the antigen.

## Results

Gliadin in ethanolic extract of wheat cultivars was assessed on the basis of its immunoreactivity with monoclonal antibody R5, using PWG gliadin as the standard.

Some differences in the content of gliadin regarding various wheat cultivars and their total protein contents could be seen on Table 1. Investigated non-bread wheat cultivars: **B, Z **and **D **are with the approximately three time lower content of immunogenic gliadin epitopes then cultivars **S, R **and **P **which are used for bread production.

**Table 1:** Gliadin content in thirteen different wheat cultivars.

No	Wheat cultivar	Wheat species	Total gliadin mg/ml extract of wheat determined on the basis of R5	Gliadin content in wheat (mg/g)	Protein content in wheat(%)	**Gliadin content/Protein content(%)**
1	R	bread	3.27	65.4	11.4	57.4

2	Z	bread	2.825	56.5	13.8	40.94

3	K	bread	2.64	52.8	12.8	41.25

4	D	durum	1.28	25.6	11.2	22.86

5	Zi	durum	1	20	11.2	17.86

6	P	bread	3.24	64.8	11.6	55.86

7	Pa	bread	2.76	55.2	12	46

8	So	bread	2.88	57.6	13.4	42.99

9	S	bread	3.54	70.8	12	59

10	B	durum	1	20	11.2	17.86

11	T	bread	2.88	57.6	12	48

12	Lj	bread	2.1	42	12	35

13	E	bread	2.52	50.4	9.6	52.5

Fifteen patients with histopathologically confirmed celiac disease were included in investigation: all of them were not on gluten-free diet (GFD).

**Figure 1:**
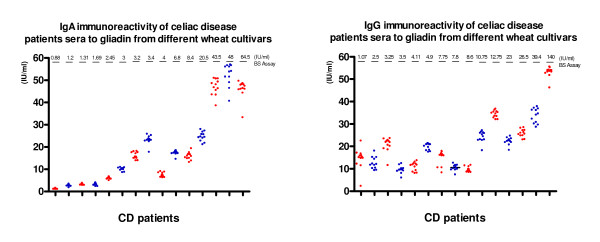
**IgA and IgG immunoreactivity of celiac patients sera to gliadin of diiferent wheat cultivars**.

Results obtained analyzing fifteen CD patients' sera before GFD, using commercial BS test, presented on Figure 1., showed the enhanced levels of anti-gliadin IgA and IgG immunity in seven and nine patients respectively. Data presented, showed that IgA immunoractyivity of CD patient's sera with gliadin isolated from tested wheat was in 3 patients more than three times higher than that obtained on BS commercial test, revealing that 10/15 CD patients had the enhanced serum antigliadin IgA antibodies.

It could be also seen that IgG immunoractivity of patient's sera with gliadin isolated from tested wheat and crude Sigma gliadin was in six patients more than three times higher than that obtained on BS commercial test, revealing that almost all analysed CD patients' sera had the enhanced levels serum antigliadin IgG antibodies.

**Figure 2:**
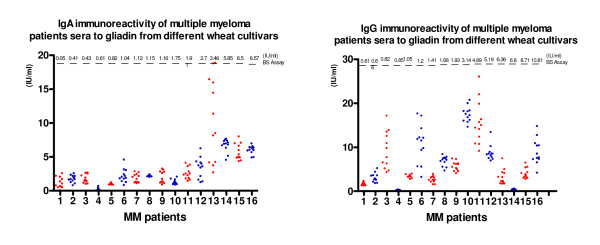
**IgA and IgG immunoreactivity of multiple myeloma patients sera with gliadin from diiferent wheat cultivar**.

The modest increase in IgA antigliadin antibodies were found by BS test in three MM patients' sera, as it could be seen on Figure 2. More then four times higher IgA immunoreactivity with gliadin isolated from local wheat was found in one patient with IgA(λ) myeloma. While the enhanced IgG immunity to gliadin in BS test was observed in one from sixteen myeloma patients, more then five times higher reactivity then that on commercial BS test was found in additional four MM patients' sera on tested wheat gliadin. It must be noted that two out from these four patients' sera did not show the enhancement in the IgG reaction with Sigma gliadin.

## Discussion

Immunity to food antigens (gliadin, cow's milk proteins, CMP) is in the centre of the attention of modern medicine [13] focused on the prevention of diseases, prevention which is based on the use of only appropriate restriction diet. Detection of the enhanced levels of the immune reactions in host, to antigen(s) present in food is from this point of view of great importance because some of premalignant conditions like monoclonal gammopathy of undetermined significance (MGUS), disappeared after the appropriate restriction diets: GFD diet [14] and CMP free diet [15]. Thus detecting the enhanced immunity to some of proteins present in food in people with some of immunological health disturbances must be followed by more detailed examination of immunogenic antigens in order to prevent some more serious diseases like NHL, or MM.

It is well known that immunomediated toxicity of gliadin is present in relatively small population of genetically predisposed individuals, with class II human leukocyte antigens HLA-DQ2 or -DQ8 which are found in nearly all celiac patients. Non-HLA genes clearly may play a role in celiac disease as well. The immunologic response to gluten includes antibody reactivity to gluten proteins and the autoantigen TG2, CD4+ T cell reactivity to gluten, increased number of intraepithelial CD8+ T cells, NK cells, and elevated levels of a number of cytokines and chemokines [16]

In recent papers an increase in the incidence of certain cancers among celiac disease patients, including non-Hodgkin lymphoma, enteropathy-associated T-cell lymphoma, small intestinal adenocarcinoma, and esophageal and oropharyngeal squamous carcinoma was reported, together with the data that strict adherence to gluten-free diet has been found to be effective at reducing the risk of some malignancies [17]. The presence of anti-gliadin and of anti-tTG imunoreactivity was found in patients with multiple myeloma too [13], as well as the anti- CMP (results not shown).

Results obtained in this work using monoclonal antibody R5, clearly show that three of investigated wheat cultivars: **B, Zi **and **D **are with the approximately three time lower immunogenic content of gliadin then cultivars **S, R **and **P**. They are in accordance with the already published data that tetraploid wheat *T. durum *do not contain the D genome, are hence already devoid of the known immunogenic sequences of α-gliadins [11]. They are also along with similar data that there is various content of immunogenic gliadin in some of wheat cultivars grown in EU countries [6].

In both patients groups IgA immunoreactivity to gliadin from different cultivars was almost homogenous and in correlation with results from commercial test (exept for one patient with IgA(λ) myeloma). But, results for IgG immunoreactivity were more frequently inhomogeneous, and especially for few MM patients they were increased and did not correlate with results obtained using Binding Site test.

Regarding durum wheat immunogenicity our results showed lower levels of humoral immunoreactivity to durum wheat gliadins than to bread wheat gliadin in CD patients and open an interesting area for further research that can elucidate whether observed differences in immunogenicity can be confirmed using peptic-tryptic gliadin digest or deaminated gliadin, as the antigen [18]. But it must be noticed that in three myeloma patients the anti-gliadin immunity was preferentially pronounced on gliadin from one tested durum wheat **Zi**, in relation to Sigma gliadin.

This set up the question is there a need to develop new standard antigen, the representative mixture of gliadin isolated from wheat species used for bread and pasta production in corresponding geographic region for ELISA diagnostic tests.

## Conclusion

Results obtained showed that there is different content of immunogenic gliadin epitopes in various species of wheat. They also point for new effort to elucidate whether more reliable data on the existence of immunoreactivity to wheat proteins in some people would be provided by testing their sera for the immunoreaction with gliadin from ethanolic extract of local wheat cultivars used for bread making in the appropriate geographic region.

## Methods

### Plant material

The experimental material consisted of ten bread **(P, R, E, So, Lj, S, T, Z, Pa**) and three durum (**D, Zi, B**) wheat cultivars developed and commercially grown in Serbia and neighbour countries. Grain samples of the bread and durum wheat cultivars were collected in 2006 from plants grown in field-trials at the Center for Agricultural and Technological Research in Zajecar, Serbia.

In the order to determine the percentage of gliadin per gram of thirteen different wheat cultivars, gliadin was isolated from flour (0.125–0.250 mesh) by two step 60% ehanolic extraction (for every sample 0,5 g of wheat flour was extracted with 5 ml of 60% ethanol with agitation over night; after that it was centrifuged 10 min on 3000 rpm, supernatant was decanted and 5 ml of 60% ethanol were added to the pallets once again for 1 hour with agitation; after centrifugation supernatants were collected). Gliadin content in wheat cultivars' ethanolic extracts, as well as in the ethanolic exract of crude Sigma, or Fluka gliadin, after appropriate dilution, was determined according to manufacturer, by commercial sandwich ELISA tests, using R5 monoclonal antibody (Ridascreen, R-Biopharm, Germany), and PWG gliadin as the antigen). Protein content of investigated wheat was determined with Kjeldahl method, based on determination of total nitrogen.

### Patients

Fifteen patients with histopathologically confirmed celiac disease were included in investigation: all of them were not on gluten-free diet (GFD). Seven had the enhanced antigliadin IgA and nine patients were with the increased levels of IgG immunoglobulins, but four out from fifteen CD patients had no enhanced levels of both anti-gliadin IgA and IgG antibodies, determined on commercial BS test.

The group of multiple myeloma patients consisted of sixteen patients with multiple myeloma, before therapy. Among them three had the enhanced IgA immunoreactivity and only one patient had the enhanced concentrations of anti-gliadin IgG antibodies determined on commercial BS test.

Informed consent was obtained from each patient and the Ethics Committee of the Institute of Oncology and Radiology of Serbia (for patients with MM) and Ethics Committee of the Clinical Center of Serbia (for patients with CD) approved the study protocol.

After determination of the concentrations of anti-gliadin IgA and IgG antibodies in tested sera by standard diagnostic ELISA Binding Site test in (IU/ml) according to manufacturer instructions, the additional non-commercial ELISA tests were done. Briefly, wells were coated with 100 μl of ehanolic extracts of gliadin isolated from mentioned wheat cultivars by two step 60% ehanolic extraction and standardized to make final quantity of gliadin (5 μg/wells) by monoclonal antibody R5 (Ridascreen, R-Biopharm, Germany). At the same way standardized 60% ethanolic extract of crude gliadin from Fluka and crude gliadin from Sigma Chemicals were used as the antigens, too. HRP labeled antibodies (Binding Site), were used as secondary antibodies. Blocker was 1% bovine serum albumin (BSA). After blocking, 100 μl of diluted (1:100) tested sera and one control serum were dispensed in appropriate wells. During the first incubation, anti-gliadin antibodies from human serum recognizing the antigen bind to it, while all unbound proteins were removed by washings. After that, purified peroxidase labeled sheep anti-human IgA, or IgG conjugates (100 μl) were added, they attach to the captured human autoantibodies, and the excess of unbound conjugate was removed by washings. The optical density (OD) of developed colour was measured on ELISA reader. Standardization of the results in repeated ELISA assays was done expressing these OD in (IU/ml) after the comparison with OD developed after the reaction of one CD control patients' serum with ethanolic extract of gliadin (Sigma) which was present in every test, and who had high anti-gliadin immunoreactivity by BS commercial test. Standardization of the results with control patient's serum in ELISA tests was done separately for both groups of analyzed patients.

## Authors' contributions

ZJ designed the study, interpreted the data and wrote the first draft and last version of the manuscript. AKR isolated gliadin from wheat, performed determination of gliadin and total protein content in various wheat, made and perform ELISA tests, presented and interpreted data and wrote the manuscript too. DD grown the wheat specimens and revised critically the manuscript and added important points to the discussion. RK, NjJ, BBN, DP and SJ enrolled the patients with celiac disease and multiple myeloma respectively, revised critically the manuscript and added important points to the discussion. JR and AN have done immunophenotyping of "M" components in myeloma patients sera, IS, IB and MDj helped with ELISA serum testing and added important points to the discussion, too. All authors approved the final draft of the manuscript.
